# Evaluation of QA software system analysis for the static picket fence test

**DOI:** 10.1002/acm2.13618

**Published:** 2022-05-15

**Authors:** Julien Boudet, Léone Aubignac, Amandine Beneux, Frédéric Mazoyer, Igor Bessieres

**Affiliations:** ^1^ Department of Physics Centre Georges François Leclerc Dijon France; ^2^ Department of Physics Hospices Civils de Lyon Pierre Bénite France; ^3^ Department of Radiotherapy Centre Hospitalier Annecy Genevois Epagny Metz‐Tessy France

**Keywords:** MLC, picket fence test, quality assurance, VMAT

## Abstract

Intensity modulation treatments are widely used in radiotherapy because of many known advantages. In this context, the picket fence test (PF) is a relevant test to check the Multileaf Collimator (MLC) performances. So this work compares and evaluates three analysis platforms for the PF used routinely by three different institutions. This study covers two linear accelerators (Linac) with two MLC types, a Millenium 120 MLC and Millenium 120 High Definition MLC respectively on a Varian Truebeam and Truebeam STx. Both linacs include an As 1200 portal imager (EPID). From a reference PF plan, MLC errors have been introduced to modify the slits in position or width (shifts from 0.1 to 0.5 mm on one or both banks). Then errors have been defined on the EPID to investigate detection system deviations (signal sensitivity and position variations). Finally, 110 DICOM‐RT images have been generated and analyzed by each software system. All software systems have shown good performances to quantify the position errors, even though the leaf pair identifications can be wrong in some cases regarding the analysis method considered. The slit width measurement (not calculated by all software systems) has shown good sensitivity, but some quantification difficulties have been highlighted regardless of the analysis method used. Linked to the expected accuracy of the PF test, the imager variations have demonstrated considerable influence in the results. Differences in the results and the analysis methods have been pointed out for each software system. The results can be helpful to optimize the settings of each analysis software system depending on expectations and treatment modalities of each institution.

## 1 INTRODUCTION

Modulated radiotherapy using Dynamic Movement of the Multileaf Collimator (DMLC)^1^, ^2^, ^3^, ^4^, ^5^ is widely used and recognized to improve target conformity and normal tissue sparing. This modulation is achieved using two techniques: intensity modulated radio therapy (IMRT) or volumetric modulated arc therapy (VMAT). Considering conventional linear‐accelerators (linacs), intensity modulation is achieved through different parameters, according to the technique used: MLC position for step‐and‐shoot (or segmental) IMRT, MLC position and speed variation for sliding window (or dynamic) IMRT and additionally, gantry rotation speed, and dose rate variation for VMAT.^6^, ^7^

The use of these complex treatment techniques requires:

1. A robust validation of the beam and MLC modeling in the treatment planning system (TPS) at commissioning.
2. Regular quality assurance (QA) control of mechanical performances of the linac.^8^, ^9^, ^10^, ^11^, ^12^
3. Patient‐Specific QA (PSQA).^13^, ^14^

Successful MLC modeling and validation comprise a crucial initial step. Subsequent regular QA of the stability of the MLC mechanical performance is required to ensure the accuracy of each leaf position during every treatment.^15^ Several authors^16^, ^17^, ^18^ have studied the impact of leaf positioning accuracy on the delivered IMRT fields pointing out an average dose delivery error up to 5% for standard localizations with a systematic MLC gap error of 1 mm. In steep dose gradient area, even for a submillimetric MLC gap error, significant dose deviations can be caused.^19^ Finally, the achievement of PSQA allows verification of the deliverability of each treatment plan.

In this context, a common test conducted to measure the positional accuracy of the MLC is the picket fence (PF) test. This test provides an assessment of the position of each MLC leaf individually and in relation to the alignment of the other leaves by using specified intervals to irradiate a series of narrow bands.^8^, ^9^

Historically, the PF test was performed with radiochromic films^9^ because of their high spatial resolution. Today, the PF test is commonly performed with the portal imager (EPID).^20^, ^21^, ^22^, ^23^ The use of this device is easier by a direct generation of a DICOM‐RT image with a submillimetric spatial resolution. For the assessment of the leaf position accuracy, a single qualitative visual inspection is not achievable. Consequently, software systems have been developed to perform a quantitative analysis of the PF DICOM‐RT image.

Three different software systems are considered in this study: Artiscan, Pylinac, and Qualimagiq. To date, no comparison between these software systems has been related to the literature. In this context, this work aims to compare and evaluate the PF test results of these three analysis platforms.

The same group of images has been analyzed by three independent institutions using each solution with their own practices. The results have been collected, compared, explained, and discussed.

## 2 METHODS

### 2.1 Linac

The entire set of data has been acquired on either a **Truebeam** with **Millenium 120 MLC** (120 MLC), or a **Truebeam STx** with **Millenium 120 High Definition MLC** (HD120 MLC), (Varian, Palo Alto, USA) linear accelerator. Each MLC is separated in two banks of 60 leaves called A and B Banks. The main difference between both MLC lies in the leaf width at the isocenter. For the 120 MLC, the leaf width is 5 mm for the 40 inner leaves and 10 mm for the 20 outer leaves. For the HD120 MLC, the leaf width is 2.5 mm for the 32 inner leaves and 5 mm for the 28 outer leaves.

Both linacs include an **As 1200** portal imager having a 43 × 43 cm^2^ sensitive matrix of 1280 × 1280 Amorphous Silicon (A‐Si) semi‐conductors with a 0.336 mm pixel resolution. The portal imager is calibrated for dosimetric measurement according to VARIAN specifications and positioned at the Source Detector Distance of 100 cm for a photon beam quality of 6 MV.

Irradiations were performed in integrated image mode for a 6 MV photon beam at the maximal dose rate (600 MU/min).

### 2.2 Reference PF

**FIGURE 1:**
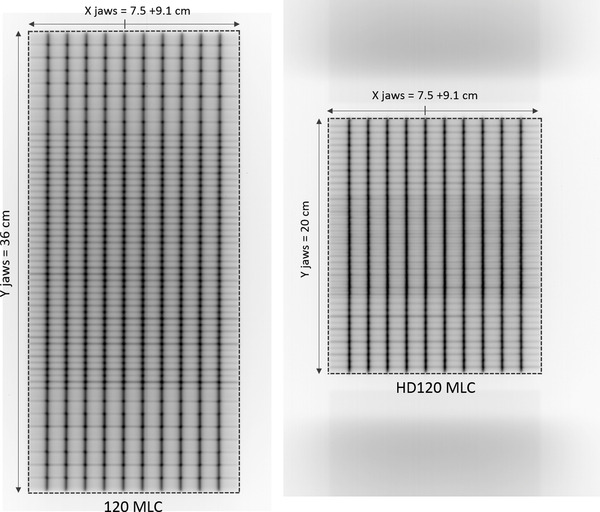
Picket fence test description for the two reference plans, the 120 Multileaf Collimator (MLC) (on the left) and the HD120 MLC (on the right)

The PF test consists in a dynamic MLC irradiation describing narrow bands (or slits) at specific and regular intervals, as shown in Figure 1.

In this study, the reference PF plan has been downloaded from the « *RapidArc QA Test Procedures and Files for TrueBeam *» package.^24^ This plan includes 10 slits (with a 1 mm nominal gap width) spaced by 15 mm. The irradiation is performed with 100 MU at a gantry and collimator position of 0°. The jaws position of the reference plan has been adjusted as following and represented on Figure 1:

1. In the inplane direction (perpendicular to leaf motions), in order to avoid a possible imager edge effect, four peripheral leave pairs have been excluded. This is only necessary for the 120 MLC and its maximum MLC field size of 40 cm. We chose to apply the same logic to the HD120 MLC to keep the same number of leaves included into the analysis. Consequently, the symmetric Y jaw aperture is 36 cm for the 120 MLC and 20 cm for the HD120 MLC.
2. In the crossplane direction (parallel to leaf motions), the X jaw aperture has been set to be 15 mm distant from the first and the last slit. Consequently, for both MLC, X1 and X2 jaws are respectively opened at 7.5 cm and 9.1 cm.

### 2.3 Analysis software systems

Three software systems capable of analyzing DICOM‐RT images acquired with the portal imager have been used in this study: Artiscan, Pylinac, and Qualimagiq (Table 1). Each system has its own type of parameters, and none are strictly equivalent. In order to simplify the reading of the paper, names have been defined for each system parameter following this nomenclature: ARTI_Name_of_Parameter, QUALI_Name_of_Parameter, and PYLI_Name_of_Parameter, respectively for each software system.

**TABLE 1:** Analysis software system description

				Parameters								
				***Position***			***Width***					
Software system	Access	Irradiation information	Template analysis placement	Algorithm	Threshold	Global slit shift	Local slit shift	Algorithm	Threshold	Slit shift	User alert	Archiving and following
Artiscan	Licensed	Yes (Dicom Tags)	Irradiated images	Center of the segment defined by a threshold	Not impacting	Relative to a reference slit (the 5th)	Relative to a reference slit (the 5th)	Distance between both intersections of a specific threshold	**Local**, from the peak value of each slit	Difference with the expected value	Yes	Yes
Pylinac	Free	None	EPID coordinates	Center of the segment defined by a threshold	Not impacting	Absolute position with EPID coordinates	Relative to its global slit position	Not returned	Not returned	Not returned	No	No
Qualimagiq	Licensed	Yes (Dicom Tags)	Irradiated images	Center of the segment defined by a threshold	Not impacting	Relative to a reference slit (the 5th)	Relative to a reference slit (the 5th)	Distance between both intersections of a specific threshold	**Global**, from the image signal of all slits	Difference with the expected value	Yes	Yes

#### 2.3.1 Artiscan V4.14.1 (Aquilab)

Artiscan^25^ is a licensed application developed by Aquilab with a specific module dedicated to the PF test and MLC performance. The default settings have been used:

Three parameters are calculated for each leaf pair:

1. The width of the slit (mm) ➔ ARTI_Width.
2. The distance between the slit of interest and the reference (mm) ➔ ARTI_Position.
3. The signal intensity of the slit given in a calibrated unit (CU) specific to Artiscan ➔ ARTI_Signal.

The position parameters are systematically calculated from a reference slit, the 5^th^ in our case and width is compared to the theoretical value.

The analysis is performed using the geometry of the irradiation taken from the DICOM information.

In Artiscan, a tolerance for each parameter can be defined and adjusted by the user. Consequently, if a value exceeds a tolerance, an alert is generated.

#### 2.3.2 Pylinac V2.0.1 (Python library)

Pylinac is a Python^26^ library developed by Kerns^27^ dedicated to Varian Linacs. The PF module is in free access and can be easily configured by the user. An advanced setting is possible with a full program access but in the context of our study, its use has been limited to the basic configuration (the default one) corresponding to a so called “typical use.”

The calculated parameters in a typical use are:

1. The MLC percentage of deviation of the peak position of each leaf pair from the average of all peak positions in the slit and regarding to a position criteria (mm) ➔ PYLI_Pass_Rate.
2. The median MLC position error (mm), considering each leaf pair and each slit ➔ PYLI_Median_Error.
3. The average distance between slits (mm) ➔ PYLI_Mean _Picket_Spacing.
4. The distance from the central beam axis to the slit (center of portal imager) (mm) ➔ PYLI_Picket_Offsets.
5. The maximum MLC position error (mm) considering each leaf pair and each slit ➔ PYLI_Max_Error.

Pylinac does not return a pass/fail result. The user defines the deviation error (0.3 mm in our case), and Pylinac returns the percentage of MLC leaf pairs that respect this deviation. The user has to determine the appropriate passing rate (typically, 100% in the institution using Pylinac).

#### 2.3.3 Qualimagiq V6.10.0 (Qualiformed)

Qualimagiq^28^ is also a licensed application with a specific module named *MLC Dyn* dedicated to the MLC performance in dynamic mode. The user can set and adjust the analyzed protocol and also select from a large number of calculated parameters. In this study, four parameters have been considered for analysis including all the leaf pairs of each slit:

1. The maximum error position (mm) ➔ QUALI_Max_Position.
2. The mean error position (mm) ➔ QUALI_Mean_Position.
3. The maximum error width (mm) ➔ QUALI_Max_Width.
4. The mean error width (mm) ➔ QUALI_Mean_Width.

The position parameters are systematically calculated from a reference slit, the 5^th^ in our case, and width is compared to the theoretical value.

The analysis is performed using the geometry of the irradiation taken from the DICOM information.

In Qualimagiq, a tolerance for each parameter can be defined and adjusted. Consequently, if a value exceeds a tolerance, an alert is generated.

### 2.4 Analysis method details

#### 2.4.1 Template positioning

The good analysis of PF images requires the extraction of the signal profiles centered under each leaf pair with the help of a rigid template. Consequently, the longitudinal positioning of the analysis template has to be done precisely. According to the software system used, two methods are applied for the template positioning. While Pylinac positions the template center using the DICOM‐RT images center, Artiscan and Qualimagiq set it by analyzing the radiative center of the Y‐jaws opening. A specific template is defined according to each MLC type.

#### 2.4.2 Slit position

**FIGURE 2:**
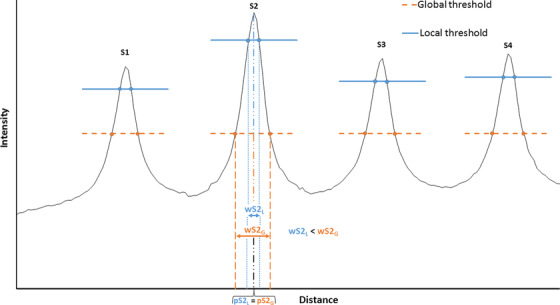
Schematic example of a drawn profile for the first four slits with a largest aperture for the 2^nd^ slit: wS2_G_ and wS2_L_ for the 2^nd^ slit width with global and local threshold, respectively; pS2_G_ and pS2_L_ for the 2^nd^ slit position with global and local threshold, respectively. In blue, the local threshold with different values applied according to the slits of interest and its peak value. In orange, the global threshold with a fixed value applied to each slit and defined according to the signal of all the slits

The slit position is typically defined by the position associated with the center of the segment defined by the intersection of the slit signal profile with a specific threshold (Figure 2).

#### 2.4.3 Slit width

The slit width is the distance between both intersections of a specific threshold applied to the signal of the slit (Figure 2).

The threshold is defined according two different methods: local and global, respectively for Artiscan and Qualimagiq. Artiscan applies a local threshold defined as a fixed percentage from each peak value making it variable according to the slit of interest. Qualimagiq applies a global threshold defined according to extrema signal on the image making it constant for all slits.

### 2.5 Altered PF

To evaluate the robustness of each analysis software system, many known errors have been inserted into the reference PF plan (Table 2). A new plan has been created for each error introduced and for each plan (reference one and modified ones). Finally, 110 DICOM‐RT images have been generated and analyzed. The different plans have been measured the same day to avoid variabilities (beam output, portal imager detection).

**TABLE 2:** Summary of the intended errors. The vertical dotted lines show the jaw edge. The slit number i is named Si and is represented by a full vertical line (with a variable thickness according to the width of the slit). Leaf pair number j is named LPj. The spaces between the slits are specified with horizontal arrow lines

**Parameters**			**Intended errors**
Reference plan		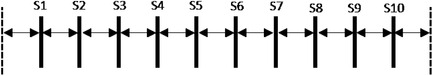	No error
Global error position	2^nd^ slit	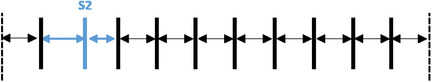	
	Central slit	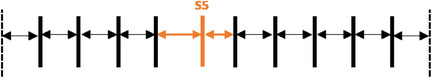	A and B Banks shift 0.1; 0.2; 0.3; 0.4; 0.5 (mm)
	All slits	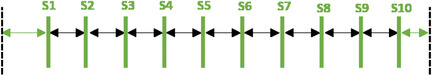	
Local error position	Leaf 30, bank A for the all slits	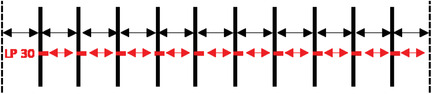	A Bank shift 0.1; 0.2; 0.3; 0.4; 0.5 (mm)
	Leaves 30–31, bank A for the all slits	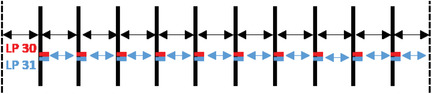	
Global error width	2^nd^ slit	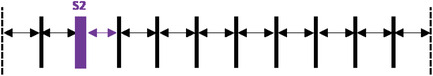	A Bank shift 0.1; 0.2; 0.3; 0.4; 0.5 (mm)
	All slits	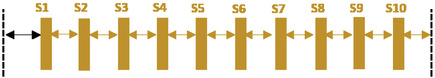	
Linac or portal imager	Signal		101; 102; 103; 104; 105 (UM)
	Rotation		0.1°; 0.3°; 0.5°; 1°; 2°
	Vertical		1; 2; 5 (mm)
	Lateral		
	Longitudinal		

Two types of error have been defined and introduced:

1. **MLC position errors** generated with an in‐house Python^26^ program:
  - Global error position: Successively, the 2^nd^ slit, the central slit, and all the slits have been shifted by five distances (0.1, 0.2, 0.3, 0.4, and 0.5 mm) ➔ 15 plans/MLC.
  - Local error position: Successively, one leaf of the A bank (number 30) and two leaves of the A bank (numbers 30 and 31) have been shifted by five distances (0.1, 0.2, 0.3, 0.4, and 0.5 mm) ➔ 10 plans/MLC.
  - Global error width: Successively, the 2^nd^ slit and all slits have been enlarged by opening the A bank by five values (0.1, 0.2, 0.3, 0.4, and 0.5 mm) ➔ 10 plans/MLC.
2. **Linac or portal imager errors** directly introduced in the TPS:
  - Signal error: to simulate a beam or detection deviation by changing the MU number (101, 102, 103, 104, and 105 MU) ➔ 5 plans/MLC.
  - Portal imager position:
    - Rotation, artificially done with five collimator positions (0.1°, 0.3°, 0.5°, 1°, and 2°) ➔ 5 plans/MLC.
    - Vertical, lateral, longitudinal positions shifted by three distances (1, 2, and 5 mm) ➔ 9 plans/MLC.

## 3 RESULTS

### 3.1 Reference PF

The analysis of the reference PF acquisition has been done with each software system.

For the slit position, the results are in agreement with the expected values for each software system reflecting the good performance of both MLC.

For the slit width, only Artiscan and Qualimagiq return dedicated values. It is interesting to notice that due to the nonflatness of the beam,^9^, ^29^ slit width variations have been observed according to the distance of the image center for both software systems. The level of these variations is more or less important according to the width calculation method of each software system. Nevertheless, these results do not reflect MLC defaults and are used as a reference baseline for the further analysis.

### 3.2 Altered PF

#### 3.2.1 Error position

##### Shift of the 2^nd^ slit

The shift of the 2^nd^ slit is detected by each software system and for both MLC.

The value of the shift is directly returned by ARTI_Position, PYLI_Picket_Offsets, and QUALI_Mean_Position (logically also by QUALI_Max_Position) parameters, respectively for Artiscan, Pylinac, and Qualimagiq.

Depending on the tolerance, an alert can be given with Artiscan and Qualimagiq. Because no tolerance is defined, Pylinac does not explicitly warn the user of the shift.

##### Shift on the 5^th^ slit (central slit)

Each software system is sensitive to the shift of the central slit for both MLC. Nevertheless, the returned values are not logical for each platform. Pylinac returns a central Py_PicketOffset value equal to the error. Artisan and Qualimagiq return a shift equal to the error on all the slits except the central one on ARTI_Position and QUALI_Mean_Position parameters. These results are explained by the analysis method that is done relatively to the central slit position that is always considered at its right position. Consequently, a shift is attributed to the other slits.

##### Shift for all slits

For both MLC, the shift of all slits impacts only the Py_PicketOffset parameter of Pylinac. With Artiscan and Qualimagiq, no shift is detected. The returned values are the same as the reference PF.

This incoherence is still due to the analysis method linked to a reference slit comparison.

#### 3.2.2 Local error position

##### Shift on the 30^th^ leaf of A bank

At first sight, the three software systems seem to detect a shift beyond an error of 0.3 and 0.1 mm respectively for the 120 MLC and the 120 HDMLC. Nevertheless, the analysis of the reference PF gives other elements to complete the analysis. Indeed, on the reference PF, one can observe that the 30^th^ leaf pair positioning is not perfect: deviation error of +0.1 mm for the 120 MLC HD and −0.1 mm for the 120 MLC. Logically for the same level of a positive introduced error, the position error for the 120 MLC is returned less important than for the MLC 120 HD. Consequently, an error of 0.1 mm can be detected with each software system on both MLC.

For Pylinac, the error is detected through PYLI_Max_Error and PYLI_Pass_Rate parameters but on the 28^th^ leaf pair instead of the 30^th^ for both MLC. This mismatch is due to the combination of the analysis method and the beam configuration. Indeed, Pylinac assumes that all the leaves are visible on the DICOM‐RT image. However in our case, jaws have been intentionally closed in the inplane direction, hiding two top and bottom leaf pairs.

This issue does not appear with Artiscan and Qualimagiq because the jaw position is taken from the RT‐plan.

For Artiscan and Qualimagiq, only with the 120 MLC, the error is always detected on the right leaf pair (the 30^th^) respectively with ARTI_Position and QUALI_Max_Position parameters.

With the HD120 MLC, the error is randomly detected on the 30^th^ or the 31^st^ leaf pair. The small size of the leaves combined with the analysis method can explain this detection default. In our case, a relative significant shift of the jaw position has been observed on the HD120 MLC linac in comparison to the small leaves size. Consequently, the analysis template has been set shifted making the analysis ROI not strictly centered on the leaf pair of interest. Consequently, depending on the shift direction, the ROI of the i±1^th^ leaf pair can receive signals from the ith leaf pair and affect the error position returned by the software system.

This situation has not been observed on the 120 MLC for Artiscan and Qualimagiq because the size of the leaves is large enough in order to be insensitive to the jaws positioning default.

Besides, whatever the software system, it is interesting to notice that the error information is systematically given for a leaf pair but never for a specific leaf or a specific bank.

##### Shift on 30^th^ and 31^st^ leaves of A bank

For Pylinac, errors are always detected through PYLI_Max_Error and PYLI_Pass_Rate parameters and always on wrong leaves (28^th^ and 29^th^) for the same reason detailed in the previous paragraph.

For Artiscan and Qualimagiq, errors are systematically detected with both MLC on the right leaf pair (30^th^ or 31^st^). The jaws configuration is the same as previously explained for the 30^th^ leaf shift only but the returned value is not impacted. The fact that two consecutive leaves have the same error introduced makes the configuration similar to a larger unique slit. So the configuration is less sensitive to the jaw calibration default, and the position error is detected either on the 30^th^ or the 31^st^ leaf pair.

#### 3.2.3 Global error width

Pylinac does not return width parameters; therefore, only results from Artiscan and Qualimagiq are presented in the following section.

##### Opening of the 2^nd^ slit

The opening of the 2^nd^ slit impacts ARTI_Signal and QUALI_Mean_Width (logically also QUALI_Max_Width) parameters, respectively, for Artiscan and Qualimagiq. While the 2^nd^ slit opening variation changes the value of ARTI_Signal, it changes the value of QUALI_Mean_Width of all slits except the 2^nd^ one. This behavior is due to the width analysis method. Indeed, Qualimagiq determines a threshold value linked to extreme signal values among all the slits. The opening of a slit causes a significant signal increase in it. Consequently, the analysis threshold will be adjusted for the slit of the extreme signal value, changing the threshold applied on the other slits (Figure 2).

Both parameters, QUALI_Mean_Width and ARTI_Signal, are sensitive to the change introduced but not specific. It is not possible to define a relation between the width of the slit opening and the returned values. Consequently, the definition of a relevant tolerance is not possible.

##### Opening of all slits

In the same way as the opening of the 2^nd^ slit, Artiscan detects the opening of all slits with the ARTI_Signal parameter.

By contrast, Qualimagiq does not detect the opening of any slits. This lack of detection is still due to the width analysis method. As the opening is the same for all the slits, the increase of the signal is proportionally the same for each slit; consequently, the applied threshold based on extreme signal values returns unchanged widths.

#### 3.2.4 Linac or portal imager

##### Signal variation

ARTI_Signal is the only parameter sensitive to a signal variation. Indeed, this parameter based on absolute CU value is mainly influenced by a beam variation or EPID performances.

##### Positioning errors

While the vertical imager positions impact the results of all platforms, only Pylinac is highlighted with the other positioning errors of portal imager. The rotation translation impacts PYLI_Max_Error with leaf identification, the lateral translation changes PYLI_Picket_Offsets and the longitudinal translation could affect the leaf identification.

So the results of the PF are mainly influenced by the mechanical performance of the portal imager.

## 4 DISCUSSION

The introduced errors have been defined in order to be representative of possible clinical deviations as follow:

1. MLC calibration default, with global shift positions of all slit.
2. Leaf gap default, with global shift widths of all slit.
3. Leaf motor default, with local shift positions.
4. Rotation acquisitions defaults:
  - MLC bank sag with global shift position (or width) of 2^nd^ slit.
  - Mechanical play of the portal imager.

Different results have been highlighted suggesting the advantages and the limitations of each software system (Table 3). Nevertheless, it is crucial to discuss the methodology of each software system in order to appreciate the quality of the result and the possible improvements.

**TABLE 3:** Software system sensitivity according to the errors. Green tick: The software system is sensitive and indicates directly the error. Blue wave: The software system is sensitive but does not indicate the error. Red cross: The software system is not sensitive to the error. NA: The software system does not return any results

			Software system sensitivity analysis 120 MLC and 120 HDMLC		
Parameters			Artiscan	Pylinac	Qualimagiq
Global error position		2nd slit	✓	≈	✓
	error	Central slit	≈	≈	≈
		All slits	✗	≈	✗
Local error position	error	Leaf 30, bank A for the all slits	✓	≈	✓
		Leaves 30–31, bank A for the all slits	✓	≈	✓
Global error width		2nd slit	≈	***NA***	≈
		All slits	≈	***NA***	✗
Linac or portal imager		Signal	Impacted	Not impacted	Not impacted
		Rotation	Not impacted	Impacted	Not impacted
		Vertical	Impacted	Impacted	Impacted
		Lateral	Not impacted	Impacted	Not impacted
		Longitudinal	Not impacted	Impacted	Not impacted

Abbreviations: HDMLC, High Definition Multileaf Collimator; MLC, Multileaf Collimator.

For the global slit position, two different analysis methods have been identified. While Pylinac calculates the distance to the center of the image (fixed position and independent from the acquisition), Artiscan and Qualimagiq calculate the distance to a reference slit (linked to the acquisition).

The first method is sensitive and specific to each combination of shift. Nevertheless, its application in Pylinac is limited because no baseline results with associated tolerance can be defined. Because of this lack of follow‐up, the user of Pylinac has to define it independently. Besides, the prerequisite of this method is to control the variation of the imager position.

Oppositely, the second method is less sensitive to the variation of the imager position. In order to be completely specific, complementary verification of the MLC isocenter has to be performed, for example an MLC collimator star‐shot^30^ or Snooker Cue test.^11^ For this purpose, Qualimagiq makes possible the inclusion of this verification to calculate the slit distance to the MLC isocenter instead of the reference slit.

Artiscan, Pylinac and Qualimagiq all perform well quantifying position deviations on global and local slits. The portal imager resolution is 0.336 × 0.336 mm^2^, but the analysis is performed with an accuracy of 0.1 mm. This level of accuracy is obtained following a method described by Mamalui‐Hunter.^31^ Despite the differences in the protocol settings, especially on the smoothing method and the threshold values definition, the calculated positions by each system are similar. This highlights the robustness of the quantification method (Figure 2).

Nevertheless wrong leaf identifications have been underlined due to the positioning of the template analysis for each software system.

In the case of Pylinac, the analysis template is placed only considering the coordinates of the imager. Consequently, no adaptation of the template is automatically done if mechanical changes appear, and wrong leaf identification can be returned. If mechanical changes are voluntary introduced (in our case, field size modification), the source code of Pylinac can be adapted in order to have the correct leaf identification. If significant mechanical uncertainties appear (for instance in the imager positioning), a bias in the leaf identification analysis might be introduced and not controlled.

In the case of Artiscan and Qualimagiq, the analysis template is placed considering the irradiated field and adjusted according to the field opening using information from the RT‐plan or the RT‐image.

Despite that the jaw calibration has been validated for a positioning in agreement with the AAPM tolerances^15^ (<1 mm), leaf misidentifications have been observed suggesting a wrong longitudinal irradiation field evaluation. Ideally the jaw has to be positioned at the edge of the first analyzed leaf pair. Nevertheless, due to a calibration uncertainty, this positioning can be inaccurate, and interleaf signal can be detected close to the edge of the irradiated field. In the case of our Artiscan and Qualimagiq results, the irradiation field opening calculation has been impacted by this additional signal, and a larger one has been calculated. The consequence is a shift in the template positioning and a possible attribution of region of interest to a wrong leaf pair. Considering this observation, the user is highly recommended to define the jaw edge position at the center of a leaf.

Consequently, the verification of the mechanical performances of the collimator and the imager (panel) is an important prerequisite to optimize the software system analysis. In parallel, the user has to consider this aspect by exploring and interpreting the results.

The robustness of each software system to quantify position deviations has been clearly demonstrated. However, the quantification of the slit width appears more complex for Artiscan and Qualimagiq (not considered by Pylinac).

As described in the reference PF results (without mechanical defaults), intensity variations in the image^9^ can change the calculated widths. While it is directly observed with the global threshold on Qualimagiq, it is not visible with the local threshold on Artiscan with ARTI_width. Initially, the local threshold seems more adapted but in fact it appears nonsensitive to infra‐millimetric errors. That is why ARTI_signal only detects the width variations.

With Artiscan, the PF image can be normalized by an open field acquisition in order to limit the beam nonflatness effects and logically optimize ARTI_Signal results. Nevertheless the local threshold method remains insensitive to this option without improving ARTI_width results.

A possible solution to improve the width quantification is to initially determine a fixed threshold according to the expected width value from a normalized PF image. This well‐defined threshold should then be applied to future PF image (normalized).

The results have demonstrated different influences of imager variations on the PF. As the imager is the detector of this test, it has to be used with a full confidence. Consequently, the regular verification of its imaging and mainly mechanical performances has to be done. In this way, Qualimagiq makes possible a pretest to quantify the mechanical play and correct it. This seems particularly relevant for the PF in gantry rotation (gravity effects) that has not been considered in our study.^32^

As discussed in this section, each software system has different advantages and drawbacks. It is useful to have them in mind in order to improve the results interpretation and to focus on reliable parameters. The optimization can be done through the protocol settings and complementary tests integrated to the software system.

The main challenge of this study is to compare many parameters that are sometimes similar but possibly calculated with different methods. Consequently, a similar PF test can be interpreted differently according to the analysis modalities.

It is highly important to think about an adapted set of tolerance that is consistent with mechanical performances and clinical objectives. For some parameters, it is difficult or impossible to define precise tolerances. In these cases, the constancy of the parameter around an initial value has to be followed in the aim of verifying the lack of global deviation. In this context, the possibility to archive and compare the results over time appears to be a precious help for the MLC performance following.

Artiscan, Pylinac, and Qualimagiq are not the only software systems available for the PF analysis. To go further, it could be interesting to reproduce this work with other systems such as *MLC QA* (IBA), ImageOwl, *RIT auto MLC* (Radiological Imaging Technology), *SNC Machine* (Sun Nuclear), or *Machine QA DoseLab* (Varian).

## 5 CONCLUSION

This work is the first comparison of the PF test analysis with three available analysis software systems. Performances and limits of each system have been evaluated for specific conditions of acquisition and settings. Introduced errors linked to clinical possible issues have been defined and applied to characterize the different software systems, to understand the analysis methods and the sensitivity.

Differences in the results and the analysis method have been pointed out for each software system. For an optimal use of each software system, it is important to have in mind the limitations of each software system.

The results can be helpful to optimize the settings of each analysis software system depending on the expectations and treatment modalities of each institution. These analysis software systems offer a fast qualitative and quantitative MLC performance QA process according to specific requirements.

## CONFLICT OF INTEREST

The authors declare no conflict of interest.

## AUTHOR CONTRIBUTIONS

*Conception and design of the work; acquisition, analysis and interpretation of data for the work; drafting the work; and final approval of the version to be published*: Julien Boudet. *Conception and design of the work; analysis of data for the work; revising the work critically for important intellectual content; and final approval of the version to be published*: Léone Aubignac and Igor Bessieres. *Analysis and interpretation of data for the work and revising the work critically for important intellectual content*: Amandine Beneux and Frédéric Mazoyer.
